# A simple and safe endoscopic technique for removing huge gastric bezoars using a looped guidewire

**DOI:** 10.1055/a-2680-5121

**Published:** 2025-08-22

**Authors:** Jae Yong Park, Kyuwon Kim, Jeongkuk Seo, Beom Jin Kim, Jae Gyu Kim

**Affiliations:** 1 37985 Division of Gastroenterology, Department of Internal Medicine, Chung-Ang University College of Medicine, Seoul, Korea (the Republic of)

Various endoscopic techniques have been applied for the removal of gastric bezoars; however, in cases of large and hard bezoars, fragmentation remains extremely challenging even with electrosurgical units or mechanical lithotriptors, often necessitating surgical intervention 1 . Although a few previous reports have described guidewire-assisted methods 1 2 3 4 , we report a very simple endoscopic technique using only a guidewire to achieve successful bezoar fragmentation ( Fig. 1 ).

**Fig. 1:**
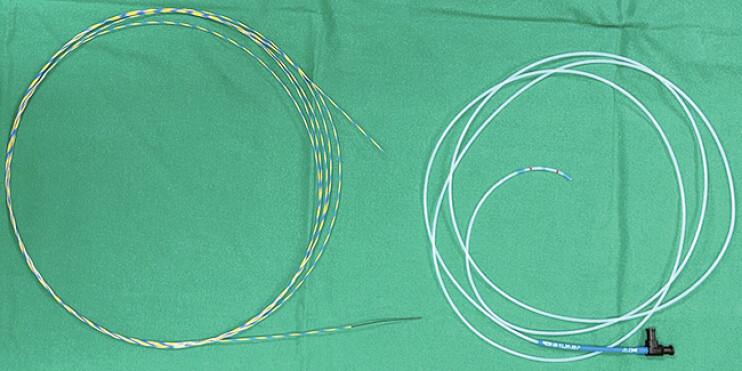
A regular guidewire (TaeWoong Medical Co.; Gyeonggi-do, South Korea) and an ERCP catheter (Jiin CNT Co.; Seoul, South Korea) used in the endoscopic procedure.

A 76-year-old man was referred for management of a giant gastric bezoar measuring 9×6 cm ( Fig. 2 **a** ), which was associated with a pressure-induced gastric ulcer ( Fig. 2 **b** ). Due to its hard consistency and large size, conventional endoscopic methods—including injection, snaring, electrocoagulation, and mechanical dissection—were unsuccessful in fragmenting the bezoar.

**Fig. 2:**
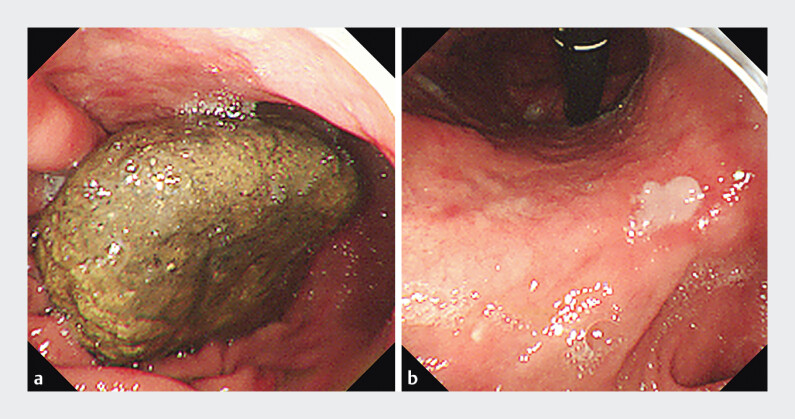
Upper endoscopic images showing the gastric bezoar. **a** A large gastric bezoar (9×6 cm) was identified in the gastric body. **b** A pressure-induced ulcer is observed at the gastric angle.

We decided to employ a dual-channel gastroscope and a standard guidewire to form a size-adjustable loop ( Fig. 3 ). The guidewire was first introduced into one working channel with the aid of a catheter, and its distal tip was then retrogradely reinserted into the catheter, which had been relocated to the second working channel ( Video 1 ). After entering the gastric lumen, the two ends of the guidewire were simultaneously adjusted through both channels to create a loop capable of capturing the bezoar ( Fig. 4 **a** ). The bezoar was fragmented by compressing it between the tightened wire loop and the distal end of the endoscope ( Fig. 4 **b** ). This maneuver was repeated until the bezoar was reduced to fragments small enough to be removed using a snare and retrieval net ( Fig. 4 **c** ). Complete removal was achieved without complications ( Fig. 4 **d** ). The whole procedure took approximately 95 minutes, including 3 minutes for preparation.

**Fig. 3:**
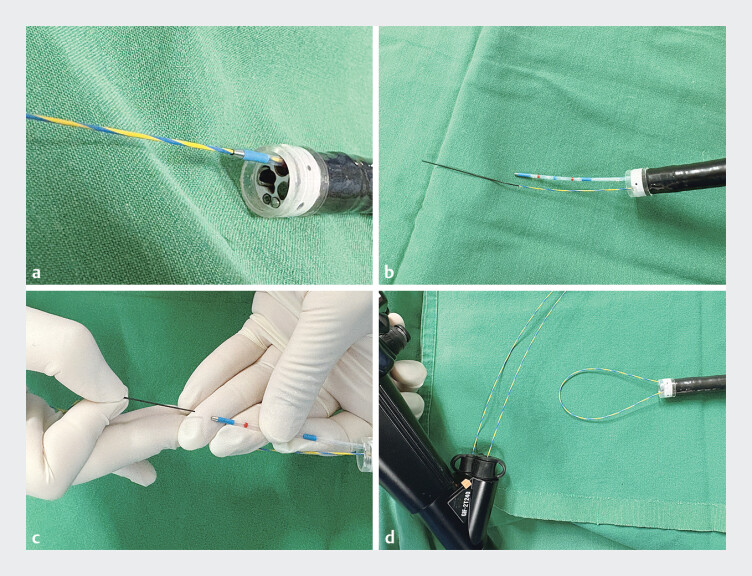
Preparation and insertion of the guidewire into a dual-channel endoscope. **a** A regular guidewire was first introduced into one working channel with the assistance of a catheter. **b** The catheter was then withdrawn and reinserted into the second working channel. **c** The distal tip of the guidewire was retrogradely inserted into the catheter positioned in the second channel, and the catheter was subsequently removed. **d** The two ends of the guidewire were adjusted through both channels to form a size-adjustable loop.

Looped guidewire technique for endoscopic fragmentation and removal of a large gastric bezoar.Video 1

**Fig. 4:**
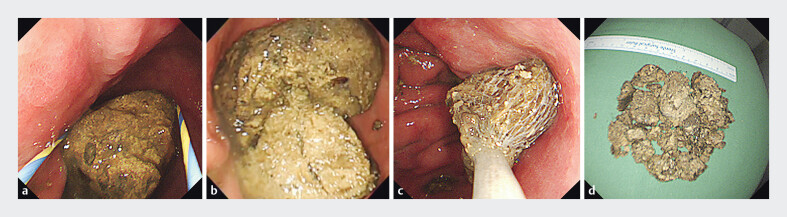
Stepwise endoscopic fragmentation and removal of a large gastric bezoar using a looped guidewire. **a** The bezoar was encircled by the looped guidewire. **b** Fragmentation was achieved by pulling both ends of the guidewire to compress the bezoar against the distal end of the endoscope. **c** Repeated fragmentation reduced the bezoar to retrievable pieces. **d** All fragments were successfully removed without complications.

This technique represents a simple, safe, and effective method for managing huge gastric bezoars using readily available equipment. It can be particularly valuable in settings with limited resources or in patients who are poor candidates for surgery.

Endoscopy_UCTN_Code_TTT_1AO_2AL

## Notes

Endoscopy E-Videos https://eref.thieme.de/e-videos
*E-Videos* is an open access online section of the journal *Endoscopy* , reporting on interesting cases and new techniques in gastroenterological endoscopy. All papers include a high-quality video and are published with a Creative Commons CC-BY license. Endoscopy E-Videos qualify for HINARI discounts and waivers and eligibility is automatically checked during the submission process. We grant 100% waivers to articles whose corresponding authors are based in Group A countries and 50% waivers to those who are based in Group B countries as classified by Research4Life (see: https://www.research4life.org/access/eligibility/ ). This section has its own submission website at https://mc.manuscriptcentral.com/e-videos .
